# Tislelizumab-associated toxic epidermal necrolysis in an esophageal cancer patient: a case report

**DOI:** 10.3389/fimmu.2025.1707956

**Published:** 2025-11-05

**Authors:** Sha Jin, Zehu Liu, Fengming Zheng

**Affiliations:** Department of Geriatrics, Hangzhou Third People’s Hospital, Hangzhou, China

**Keywords:** tislelizumab, esophageal cancer, toxic epidermal necrolysis, PD-1 inhibitor, immune-related adverse events, case report

## Abstract

**Background:**

Tislelizumab, a humanized IgG4 anti-programmed cell death 1 (PD-1) monoclonal antibody approved in China in 2019 for advanced solid tumors such as esophageal cancer, functions by blocking the PD-1/PD-L1 pathway to reactivate anti-tumor immunity. Common adverse reactions include fever and rash; however, toxic epidermal necrolysis (TEN)—a rare, life-threatening drug hypersensitivity reaction—is reported in fewer than 0.1% of patients receiving PD-1 inhibitors, with limited real-world evidence specifically linking it to tislelizumab.

**Case presentation:**

A 70-year-old male with esophageal squamous cell carcinoma received two cycles of neoadjuvant therapy (nab-paclitaxel, cisplatin, and tislelizumab 200 mg) followed by partial esophagectomy. On day 86 after the first tislelizumab infusion, he developed a diffuse rash progressing to skin exfoliation, vesiculation, and a positive Nikolsky sign, leading to a diagnosis of TEN. Upon admission, his SCORTEN was 3 (predicting 35% mortality) and ALDEN score was 5, indicating a probable association with tislelizumab. Management included intravenous methylprednisolone, immunoglobulin, topical treatments, and nutritional support. The patient achieved complete recovery two months after symptom onset.

**Conclusion:**

This case illustrates that tislelizumab can induce TEN after a prolonged incubation period (86 days in this instance). It underscores the importance of vigilant monitoring of skin and mucous membranes during treatment, early recognition and intervention, and adequate glucocorticoid dosing in managing this serious immune-related adverse event, offering valuable clinical insight for oncologists.

## 1 Introduction

Tislelizumab is a humanized immunoglobulin G4 (IgG4) monoclonal antibody designed to target the programmed cell death protein 1 (PD-1) receptor. It received regulatory approval in China in 2019 and is currently indicated for the treatment of several advanced solid malignancies, including esophageal carcinoma (1). Mechanistically, tislelizumab binds with high specificity to the PD-1 receptor, effectively inhibiting its engagement with the ligands PD-L1 and PD-L2. This blockade mitigates PD-1-mediated immunosuppressive signaling commonly exploited by tumor cells to evade immune surveillance (2). As a result, T-cell function is restored, enabling the immune system to recognize and attack tumor cells more effectively (3). In clinical practice, the most frequently observed adverse events associated with tislelizumab include fever, fatigue, rash, pruritus, cough, anorexia, nausea, vomiting, and diarrhea (4).

Toxic epidermal necrolysis (TEN) is a rare but life-threatening cutaneous adverse reaction, predominantly drug-induced, characterized by extensive keratinocyte apoptosis leading to widespread epidermal detachment, erosions, and mucosal involvement. As a severe mucocutaneous syndrome, TEN represents a medical emergency with high morbidity and mortality, often stemming from a hypersensitivity reaction to medications (5). Diagnosis is primarily clinical, supported by histopathological findings, and requires prompt drug withdrawal and multidisciplinary management. In this report, we describe a case of TEN that arose following tislelizumab therapy in a patient diagnosed with esophageal cancer, highlighting its clinical course, management, and outcome.

## 2 Case report

A 70-year-old male patient was admitted to our institution on April 22, 2025, presenting with a chief complaint of “dysphagia lasting 7 months, and status post esophageal cancer surgery performed 1 month prior.” The patient’s medical history indicated that a gastroscopic examination conducted on January 6, 2025, had identified a polypoid elevated lesion located 30 cm from the incisors, exhibiting surface roughness, superficial erosion, and a tendency to bleed easily. Histopathological analysis of the biopsy specimen confirmed the diagnosis of squamous cell carcinoma. As part of a neoadjuvant treatment strategy, the patient received two cycles of chemotherapy combined with immunotherapy at an external hospital on January 12 and February 10, 2025. The therapeutic regimen consisted of nab-paclitaxel (180 mg administered on days 1 and 8), cisplatin (40 mg on days 1–3), and tislelizumab (200 mg on day 1) per cycle. Subsequently, on March 25, 2025, the patient underwent a partial esophagectomy via a three-incision approach (cervical, thoracic, and abdominal) under general anesthesia. Postoperative management included anti-infective prophylaxis with cefoperazone-sulbactam(2.0g intravenously infused every 12 hours, from March 26, 2025 to April 9, 2025), as well as supportive therapies such as gastric protection and fluid resuscitation. The patient had no previous history of allergy to cephalosporins or penicillins. Preoperatively, he was hospitalized for pulmonary infection and received cefoperazone-sulbactam without any allergic reactions.

**Figure 1:**
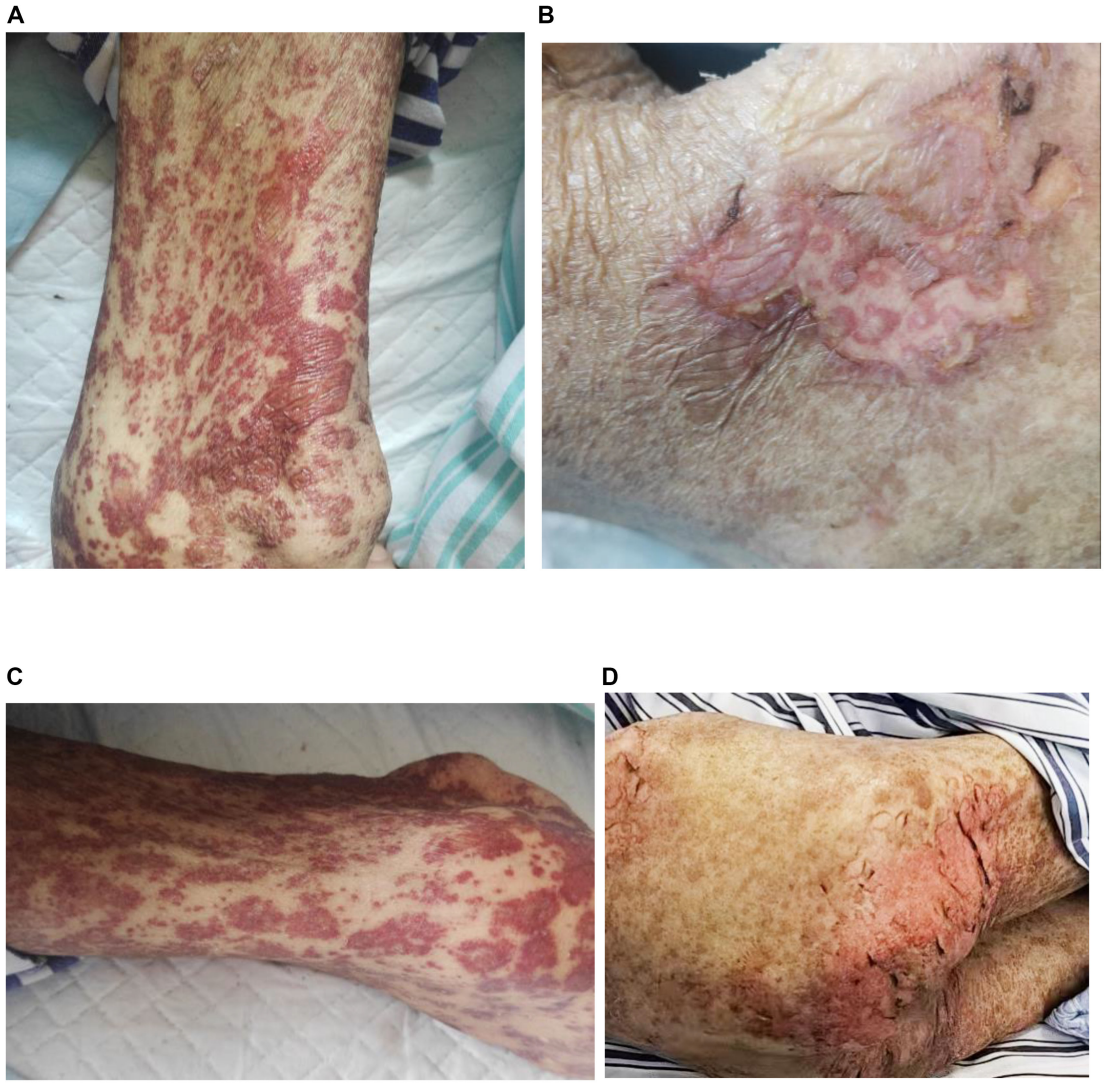
Dermatological presentation of toxic epidermal necrolysis induced by tislelizumab; **(A)** Rash and blisters on the arm; **(B)** skin detachment on the waist; **(C)** rash on the leg; **(D)** skin detachment on the buttocks.

On April 8, 2025, the patient began developing a generalized scattered rash, which progressively worsened. The cutaneous manifestations evolved to include extensive exfoliation and erosions affecting the buttocks and hips, along with widespread dark-purple erythema, vesicles, and bullae distributed across the trunk and extremities. These lesions were associated with significant pruritus and pain. Mucosal involvement was noted, and a positive Nikolsky sign was observed (**Figures 1**), supporting the diagnosis of toxic epidermal necrolysis (TEN). Initial management, initiated on April 10, included intravenous methylprednisolone 40 mg once daily to attenuate the inflammatory response. Between April 16 and 20, the patient received intravenous immunoglobulin (IVIG) at a dose of 2.5 g every 12 hours. Following modest clinical improvement, the patient was transferred to our hospital for specialized multidisciplinary care.

**Figure 2:**
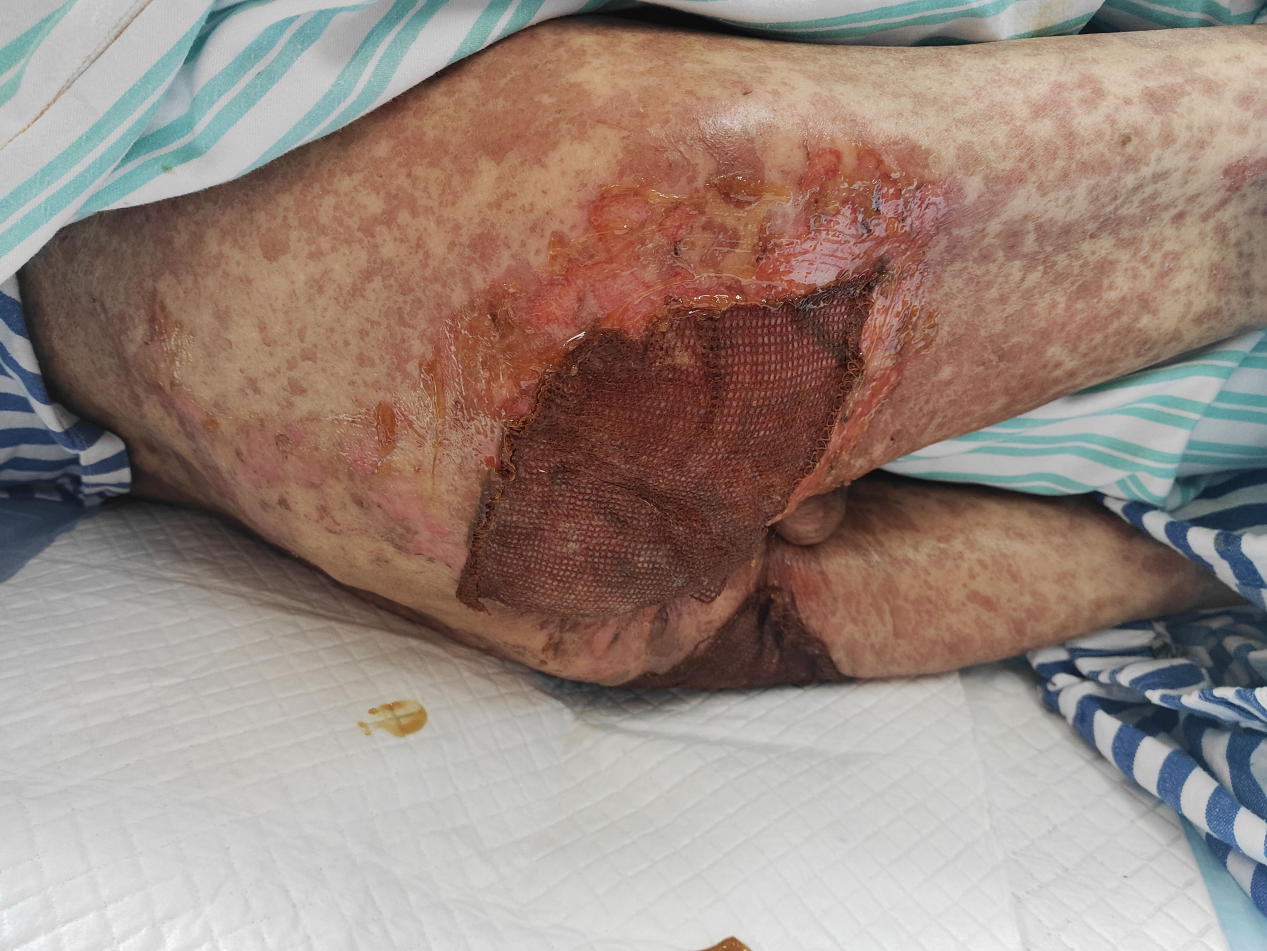
Silver-ion gauze and moist exposed burn ointment were applied to the denuded skin on the buttocks.

**Figure 3:**
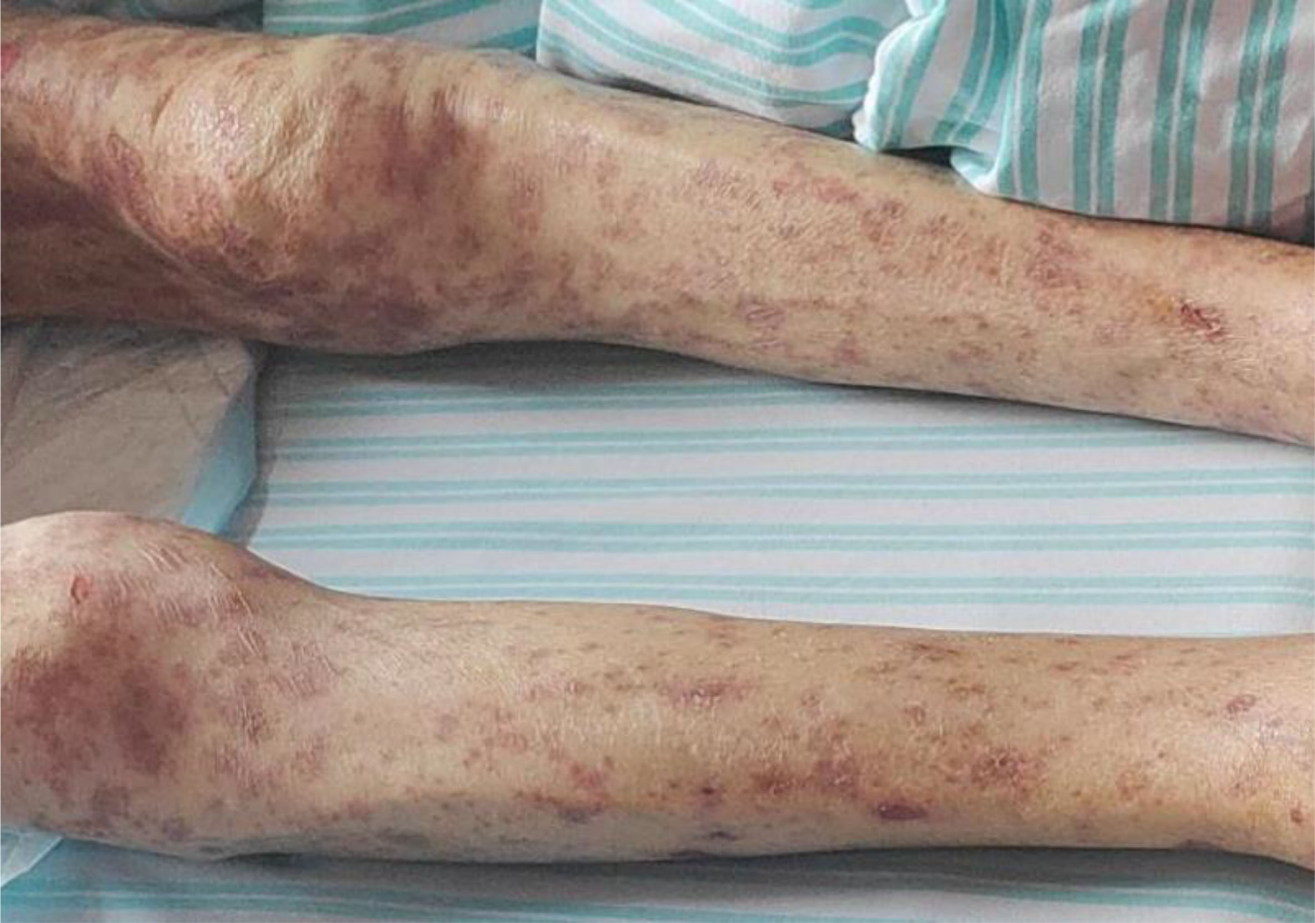
Imaging of skin following resolution of toxic epidermal necrolysis.

On admission, the patient’s condition was assessed using the SCORTEN (Severity-of-Illness Score for Toxic Epidermal Necrolysis) scale, which yielded a score of 3. This score incorporated advanced age (>40 years), underlying malignancy, and mucosal involvement affecting more than 10% of the total body surface area, corresponding to an estimated mortality risk of 35%. Furthermore, the ALDEN (Algorithm of Drug Causality for Epidermal Necrolysis) score was applied to evaluate drug causality, resulting in a score of 5 for tislelizumab—indicating a “ Very probable “ association with TEN. Cefoperazone-sulbactam had an ALDEN score of 3.During hospitalization, the patient was continued on intravenous methylprednisolone at a reduced dose of 20 mg daily for 5 days, with subsequent transition to oral methylprednisolone 8 mg once daily. The treatment also included comprehensive nutritional support, electrolyte management, and topical wound care involving the application of Moist Exposed Burn Ointment to affected areas followed by coverage with silver-ion impregnated gauze (**Figure 2**). Notable improvement in skin and mucosal lesions was observed within one week of admission (**Figure 3**). Glucocorticoids were gradually tapered and eventually discontinued. The patient achieved complete clinical recovery two months after the initial onset of symptoms,the skin has completely healed without active lesions, with only mild hyperpigmentation remaining.

The patient reported satisfaction with the treatment outcome, noting steady improvement in skin symptoms after initiating glucocorticoids and IVIG. They expressed relief at complete lesion resolution after 2 months, with no significant impact on daily activities during recovery.

**Figure 4:**
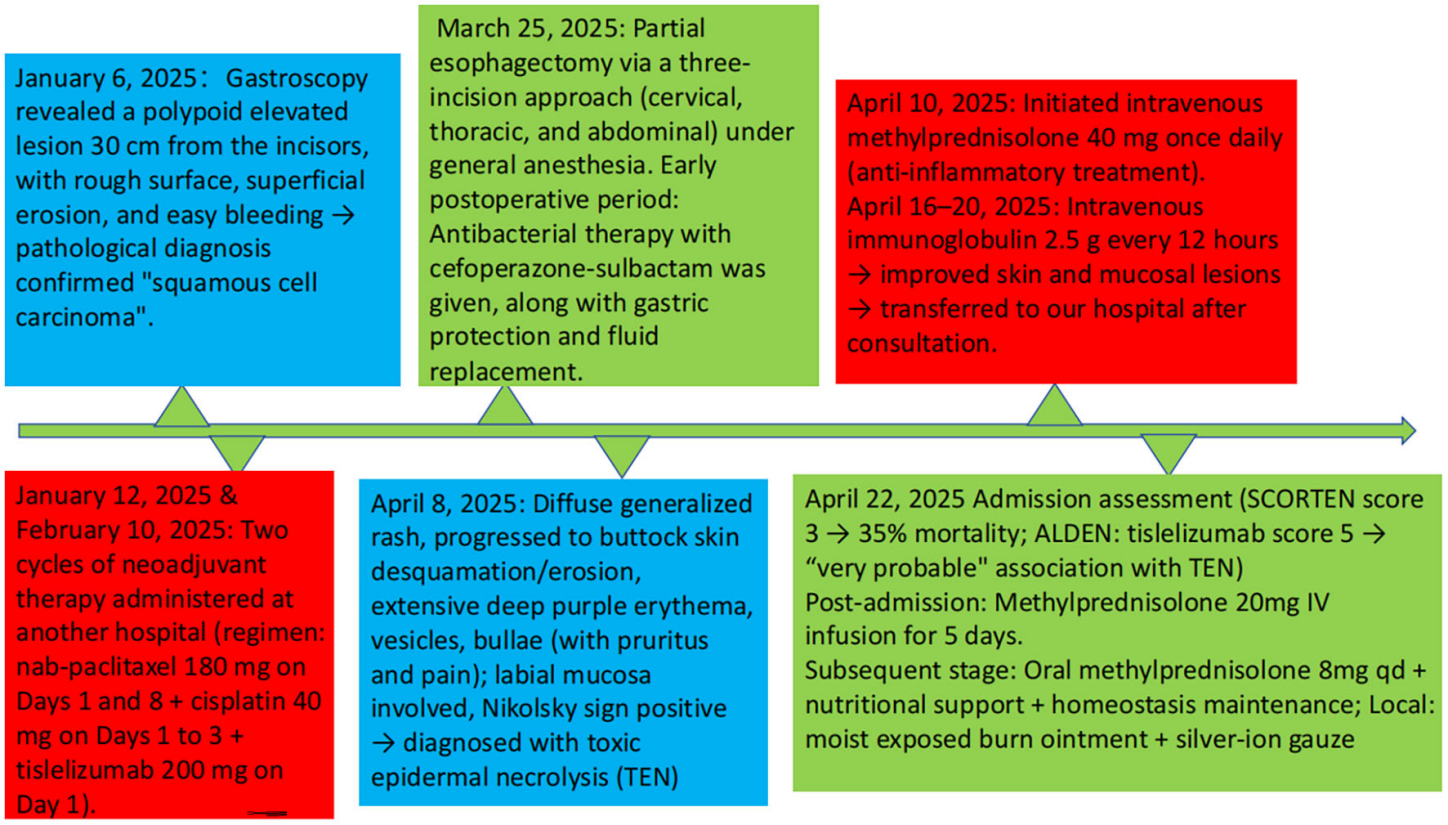
Graphical overview of the patient’s treatment timeline.

The patient presented with a rash and blisters 86 days following the first dose of tislelizumab (57 days after the second dose), which progressed rapidly to extensive epidermal detachment. This condition was deemed to be associated with the aforementioned biological agent. The patient’s symptoms showed significant improvement after administration of glucocorticoids, topical dermatological agents, and intravenous immunoglobulin. The timeline of the patient’s treatment is illustrated in **Figure 4**.

## 3 Discussion

Immune checkpoint inhibitor therapy has emerged as the standard treatment for esophageal squamous cell carcinoma (ESCC) (6). Recent clinical trials have demonstrated that perioperative immune checkpoint inhibitor combined with chemotherapy for esophageal cancer can improve overall survival and reduce mortality rates (7). Tislelizumab, a PD-1 inhibitor, exerts anti-tumor effects by blocking the PD-1/PD-L1 pathway and is widely used in the management of multiple malignant tumors. Clinical evidence has shown that PD-1 inhibitors can induce immune-related cutaneous adverse events (irCAEs), among which toxic epidermal necrolysis (TEN) is a rare yet life-threatening subtype with an incidence of less than 0.1% (8). The underlying mechanism of this association is presumably linked to drug-induced excessive immune activation: upon administration of PD-1 inhibitors, the inhibition of T cells is alleviated, leading some T cells to erroneously target the body’s own epidermal cells and cause immune-mediated damage (9). The RATIONALE-306 study reported that 97% of patients in the tislelizumab plus chemotherapy arm experienced treatment-related adverse events (TRAEs); while cutaneous toxicities including stomatitis and pruritus were noted, no cases of TEN were observed (7).

TEN is an immune-mediated type IV hypersensitivity reaction, most frequently induced by drug hypersensitivity, and identification of the causative agent is critical. The patient developed a rash and blisters 86 days after the first administration of paclitaxel + cisplatin + tislelizumab (57 days after the second cycle), which progressed rapidly to extensive epidermal detachment. Meanwhile, the patient had received cefoperazone-sulbactam prior to the onset of symptoms. Although there have been previous reports of TEN induced by this drug, the patient had no prior history of allergies to cephalosporins or penicillins, and no rash was observed when he was administered the same drug before surgery.Cutaneous reactions associated with paclitaxel and cisplatin are typically mild, manifesting as mild rash and pruritus, and no cases of TEN induced by these two drugs have been documented in domestic or international databases. In contrast, 12 case reports of TEN induced by tislelizumab have been published (10), and the incubation period in this case is the longest among all reported cases to date. Combined with the assessment of the ALDEN score, it is concluded that the patient’s TEN is highly associated with tislelizumab.

PD-1 inhibitors exert anti-tumor effects by relieving T-cell “immune brake” via the PD-1/PD-L1 pathway, yet may induce “immune dysregulation” that causes auto-tissue injury (11). For TEN induction, tislelizumab may act through two key pathways:(1) Aberrant T-cell activation: Following PD-1 blockade, effector T cells—particularly cytotoxic CD8^+^ T cells—undergo excessive proliferation, infiltrate the skin, and release perforin, granzyme, and other mediators to directly damage epidermal cells (12); (2) Cytokine storm: Activated T cells and innate immune cells (e.g., macrophages) secrete massive pro-inflammatory cytokines (e.g., TNF-α, IFN-γ), generating a “cytokine storm” that further amplifies epidermal cell apoptotic signals (13).Unlike traditional drugs (e.g., cefoperazone, which induces immediate hypersensitivity via hapten-carrier complexes), PD-1 inhibitors exhibit delayed and persistent immunomodulatory effects (11). This explains the 86-day TEN latency observed in this case, as cumulative immune imbalance requires time to reach the threshold for TEN induction. Immunotherapies such as PD-1 inhibitors exhibit more complex mechanisms, which involve sustained immune activation. Traditional methods, including the ALDEN tool, have limitations in determining drug causality (14).

Toxic epidermal necrolysis (TEN) requires multi-faceted management centered on controlling immune-mediated inflammation, preventing infection, and supporting organ function. Current guidelines recommend the following key measures: (1)Permanent discontinuation of the implicated immune checkpoint inhibitor (such as tislelizumab) to eliminate ongoing immune activation (15). (2)High-dose glucocorticoids (1–2 mg·kg^-^¹·d^-^¹ methylprednisolone or equivalent) as first-line anti-inflammatory therapy to suppress T-cell activation and cytokine release (13, 16). (3)Early administration of intravenous immunoglobulin (IVIG; 400 mg·kg^-^¹·d^-^¹ for 3–5 days) in moderate-to-severe cases. IVIG modulates the immune response by neutralizing cytokines, blocking Fc receptors, and interfering with T-cell receptor signaling (17). (4) Comprehensive supportive care, including specialized wound management and nutritional support, to facilitate re-epithelialization and reduce complications (15). In this case, the initial glucocorticoid dose (≈0.8 mg·kg^-^¹·d^-^¹) was below the recommended range, which may have contributed to suboptimal early disease control. The subsequent clinical improvement following IVIG underscores the importance of combined immunomodulation—particularly in PD-1 inhibitor–associated TEN, where multisystem immune dysregulation often necessitates a multi-targeted approach.

## 4 Conclusion

This case highlights that tislelizumab can induce TEN with an incubation period of up to 87 days. During tislelizumab treatment, close monitoring is imperative, with special attention to the patient’s cutaneous and mucosal status. If abnormalities occur, vigilance for TEN is warranted, and prompt intervention should be initiated to ensure adequate glucocorticoid dosing. In conclusion, tislelizumab-associated TEN in the treatment of esophageal cancer, although rare, constitutes a life-threatening adverse event.

## Data Availability

The original contributions presented in the study are included in the article/supplementary material. Further inquiries can be directed to the corresponding author.
